# Wnt/β-Catenin Regulates the Activity of Epiprofin/Sp6, SHH, FGF, and BMP to Coordinate the Stages of Odontogenesis

**DOI:** 10.3389/fcell.2016.00025

**Published:** 2016-03-30

**Authors:** Maitane Aurrekoetxea, Igor Irastorza, Patricia García-Gallastegui, Lucia Jiménez-Rojo, Takashi Nakamura, Yoshihiko Yamada, Gaskon Ibarretxe, Fernando J. Unda

**Affiliations:** ^1^Department of Cell Biology and Histology, Faculty of Medicine and Dentistry, University of the Basque Country UPV/EHULeioa, Spain; ^2^Center of Dental Medicine, Institute of Oral Biology, University of ZurichZurich, Switzerland; ^3^Division of Molecular Pharmacology and Cell Biophysics, Department of Oral Biology, Graduate School of Dentistry, Tohoku UniversitySendai, Japan; ^4^Laboratory of Cell and Developmental Biology, National Institute of Dental and Craniofacial Research, National Institutes of HealthBethesda, MD, USA

**Keywords:** Wnt/β-catenin, tooth development, GSK-3, BIO-culture, Epiprofin/Sp6, odontogenesis

## Abstract

**Background:** We used an *in vitro* tooth development model to investigate the effects of overactivation of the Wnt/β-catenin pathway during odontogenesis by bromoindirubin oxime reagent (BIO), a specific inhibitor of GSK-3 activity.

**Results:** Overactivating the Wnt/β-catenin pathway at tooth initiation upregulated and ectopically expressed the epithelial markers *Sonic Hedgehog (Shh), Epiprofin (Epfn), and Fibroblast growth factor8 (Fgf8)*, which are involved in the delimitation of odontogenic fields in the oral ectoderm. This result indicated an ectopic extension of the odontogenic potential. During tooth morphogenesis, *Fibroblast growth factor4 (Fgf4), Fibroblast growth factor10 (Fgf10), Muscle segment homeobox 1 (Msx-1), Bone Morphogenetic protein 4 (Bmp4), and Dickkopf WNT signaling pathway inhibitor 1 (Dkk-1)* were overexpressed in first molars cultured with BIO. Conversely, the expression levels of *Wingless integration site 10b (Wnt-10b)* and *Shh* were reduced. Additionally, the odontoblast differentiation markers Nestin and *Epfn* showed ectopic overexpression in the dental mesenchyme of BIO-treated molars. Moreover, alkaline phosphatase activity increased in the dental mesenchyme, again suggesting aberrant, ectopic mesenchymal cell differentiation. Finally, Bmp4 downregulated *Epfn* expression during dental morphogenesis.

**Conclusions:** We suggest the presence of a positive feedback loop wherein *Epfn* and β*-catenin* activate each other. The balance of the expression of these two molecules is essential for proper tooth development. We propose a possible link between Wnt, Bmp, and Epfn that would critically determine the correct patterning of dental cusps and the differentiation of odontoblasts and ameloblasts.

## Introduction

The mouse tooth is a good model for the study of regulatory pathways involved in cell differentiation, proliferation and organogenesis. Mouse tooth development begins around embryonic day 10.5 (E10,5) with a local thickening and oral epithelial invagination. Continuation of this invagination process results in the formation of epithelial tooth buds at E12.5–13.5. The mesenchymal cells condense around the buds, and after the bud stage, the tooth germ progresses to the cap (E14.5), early bell (E17.5) and late bell (E19.5) stages. From the bell stage onwards, the epithelial cells in contact with dentin differentiate into the enamel-producing ameloblasts (Thesleff and Hurmerinta, 1981; Martin et al., 1998; Ruch, 1998), and the dental papilla cells near the epithelium polarize and differentiate into dentin-secreting odontoblasts. At approximately E12.5, the potential to induce tooth formation is transferred from the dental epithelium to the dental mesenchyme (Mina and Collar, 1987; Lumsden, 1988). However, the exact molecular determinants of this odontogenic potential have not yet been completely elucidated.

There are many signaling pathways involved in different stages of dental development. The Fibroblast growth factor (FGF), Sonic hedgehog (SHH), Wingless integration site (Wnt), and Transforming growth factor β (TGFβ) superfamilies are essential in tooth development (Jernvall and Thesleff, 2000; Thesleff and Mikkola, 2002). Wnt/β-catenin signaling is highly conserved in vertebrates, and the precise control of this pathway is required for normal tooth development. Wnt/β-catenin is regulated by the activity of the serine/threonine kinase GSK-3, which physically interacts with other proteins, including Axin and Adenomatous Polyposis Coli (APC). In the absence of Wnt signaling, cytoplasmic β-catenin is phosphorylated by this complex and is targeted for degradation by the ubiquitin-proteasome system (Seidensticker and Behrens, 2000). Binding of Wnt ligands to Frizzled (FZ) receptors and low-density lipoprotein-related protein (LRP) family co-receptors activates intracellular Disheveled (DVL) proteins, eventually leading to inhibition of the destruction complex. Therefore, binding of Wnt ligands to their receptors allows cytoplasmic β-catenin accumulation, nuclear translocation, and transcriptional activation by complexes of β-catenin and LEF/TCF transcription factor family members (Logan and Nusse, 2004). In the mouse, active Wnt/β-catenin signaling occurs throughout the different stages of tooth development, performing different roles in each stage (Liu and Millar, 2010; Lohi et al., 2010). SomeWnt protein inhibitors, such as Dickkopf-1 (Dkk1), bind to LRP5/6 coreceptors and thus block transmission of Wnt signals (Andl et al., 2002). A recent work has identified LRP6, a co-receptor in the canonical Wnt/β-catenin signaling cascade, contributing to the etiology of non-syndromic autosomal-dominant oligodontia (Massink et al., 2015).

Loss of balance in the activation of the Wnt pathway produces serious problems in dental development. Increased Wnt/β-catenin signaling in conditional knockout mice results in numerous abnormal dental epithelial invaginations and supernumerary tooth formation, ectopic incisor and molar development, and alterations in the differentiation of ameloblasts and odontoblasts and dental mineralization (Järvinen et al., 2006; Thesleff, 2006; Wang et al., 2009; Ahn et al., 2010; Kim et al., 2011). Conversely, either genetic deletion of β-catenin or overexpression of Dkk1 results in complete inhibition of tooth development (van Genderen et al., 1994; Liu et al., 2008; Chen et al., 2009). In humans, mutations in Wnt10a lead to congenital hypodontia or tooth agenesis that can be either isolated (Kantaputra and Sripathomsawat, 2011) or associated with ectodermal dysplasia (Adaimy et al., 2007).

Growth factors and signaling molecules, such as Wnt, Fgf, Shh, and Bmp, are interconnected in dental development. In particular, *Fgf4* has been described as a downstream target for Lef1 and the Wnt pathway in early tooth primordia (Kratochwil et al., 2002). Other members of the FGF family, such as *Fgf9* and *Fgf10*, have also been associated with the Wnt pathway during odontogenesis (Cobourne and Sharpe, 2010). FGF plays essential roles in multiple biological processes including cellular proliferation, differentiation and survival (Unda et al., 2000; Barrientos et al., 2008; D'Andrea et al., 2009). It has also been reported that dental epithelial *Shh* expression is induced by mesenchymal FGFs (Kratochwil et al., 1996). Likewise, Wnt signaling in the dental mesenchyme is also essential for inducing *Shh* expression in the dental epithelium (Fujimori et al., 2010). *Shh* activation is a good indicator of development of the dental epithelium because in early stages, *Shh* is specifically expressed in the oral ectodermal regions where future incisors and molars will be formed (Dassule and McMahon, 1998). Additionally, members of the BMP protein family are expressed from the beginning of odontogenesis and play crucial roles in the transition from the bud stage to the cap stage and in terminal dental cell differentiation (Wang et al., 2004; Jiang et al., 2013).

Epiprofin/Specificity Protein 6 (Epfn) is a Krüppel-like family (KLF) transcription factor that is critically involved in tooth morphogenesis and ameloblast and odontoblast differentiation. However, its mechanism of action is still not fully understood. During odontogenesis, *Epfn* expression is restricted to the dental epithelium of developing molars and incisors from the bud stage to the terminal bell stage. At the late bell stage, *Epfn* is highly expressed in the preameloblastic inner enamel epithelium, and at that time, its expression also extends to the dental papilla-derived odontoblasts (Nakamura et al., 2004). The phenotype of *Epfn*-knockout mice reveals profound alterations in tooth morphogenesis and differentiation, as well as severe hyperdontia featuring supernumerary incisor and molar teeth (Nakamura et al., 2008). Our previous studies suggest that Epfn enhances canonical Wnt/β-catenin signaling in the developing dental pulp mesenchyme, a condition that promotes the activity of other downstream signaling pathways, such as BMP signaling, that are fundamental for cellular induction and ameloblast differentiation (Jimenez-Rojo et al., 2010; Ibarretxe et al., 2012). Recent studies have shown a role for Epfn in keratinocyte differentiation by reducing E2F transactivation and inducing *Notch1* expression (Nakamura et al., 2014). In addition, Epfn plays a critical role in the differentiation of inner enamel epithelium into ameloblasts. A genetic linkage between a 2-bp insertional mutation of Sp6 gene and the amelogenesis imperfecta phenotype in rats has been found. Thus, it has been proposed the addition of Sp6 mutation as a new molecular diagnostic criterion for the autosomal recessive amelogenesis imperfecta patients (Muto et al., 2012).

The aim of this work was to separately study the effects of overactivating Wnt/β-catenin signaling during each of the different stages of odontogenesis and then to relate these changes to the expression of *Epfn* and of other downstream signaling effectors such as *Bmp, Fgf*, and *Shh*. Our model consists of transient application of BIO, a highly potent, selective, reversible, and competitive inhibitor of GSK-3 (Meijer et al., 2003), to hyperactivate Wnt/β-catenin in organotypic cultures of isolated branchial arches and first molars at various developmental stages. This allows the evaluation of the effect of the Wnt/β-catenin signaling cascade during odontogenesis in the absence of interference related to overactivation/inhibition of this pathway during other periods of dental development. We analyzed the effects of Wnt/β-catenin hyperactivity on dental mesenchymal proliferation and odontoblast and ameloblast differentiation. In addition, we investigated the relationship between the canonical Wnt pathway and other signaling effector molecules involved in odontogenesis, specifically Fgf4, Fgf9, Shh, Wnt10b, Dkk1, Bmp4, and Epfn.

## Experimental procedures

### Chemicals

(2′Z,3′E)-6-bromoindirubin-3′-oxime (BIO; Calbiochem) is a cell-permeable bis-indolo(indirubin) compound that acts as a highly potent, selective, reversible, and ATP-competitive inhibitor of GSK-3.

1-Methyl-BIO (MetBIO; Calbiochem) is a cell-permeable N-methylated analog of BIO that serves as a relevant inactive control of kinase activation. BIO and MetBIO were dissolved in dimethyl sulfoxide (DMSO, SigmaAldrich) and diluted in culture media at 20 μM for *in vitro* experiments.

### Embryonic tooth culture

Mouse first branchial arches at E11.5 and first lower molars at three stages of tooth development (E14.5, E17.5, and E19.5) were dissected by microsurgery with scalpels, in Hank's buffered saline solution (Gibco) under a stereomicroscope. Teeth were placed on hydrophilized polytetrafluoroethylene (PTFE) Millicell^TM^ filters (0.4 μm diameter, Millipore) and cultured in 1 ml Roswell Park Memorial Institute (RPMI) medium (Gibco) supplemented with 0.18 mg ascorbic acid, 2 mM L-glutamine (Gibco), 0.1 mg kanamycin (Thermo-Fisher Scientific)and 20% fetal bovine serum (Biochrom), at 37°C and 5% CO_2_. Explants were treated with BIO and MetBIO (control) at 20 μM for 24 or 48 h or for 4 or 6 days. E19.5 teeth were not maintained in culture for more than 4 days *in vitro* due to size-related limitations on nutrient and oxygen exchange. The culture medium was changed every 2 days until the end of the culture. Some explants were cultured with 10 μM 5-bromo-2′-deoxyuridine (SigmaAldrich) for 2 h. Samples were processed for histology, *in situ* hybridization or immunofluorescence. The experiments with mice have been approved by the ethic committee of the University of the Basque Country, UPV/EHU (346/2014/UNDA).

### Histology and immunofluorescence

Tooth cultures were fixed overnight at 4°C in 4% paraformaldehyde (SigmaAldrich). Subsequently, they were cryoprotected overnight at 4°C in 30% sucrose and then embedded in OCT (Tissue-Tek) and stored at −80°C until use. Frozen sections (15 μm) were obtained using a cryostat (Shandon) and post-fixed in 4% paraformaldehyde for 10 min. Some sections were stained with hematoxylin-eosin or permeabilized using 1% Triton X-100 (SigmaAldrich) in PBS (SigmaAldrich) and blocked with 10% fetal bovine serum for 1 h at room temperature. These samples were incubated overnight at 4°C with the following antibodies:mouse anti-bromodeoxyuridine-fluorescein monoclonal antibody 1:200 (Roche 11202693001), rabbit anti-cleaved Caspase3 1:200 (Asp175; Cell Signaling) anti-Nestin 1:100 (ab5968; Abcam) and anti-Amelogenin 1:200 (ab59705 Abcam) antibodies. The secondary antibody used was Alexa Fluor 488-conjugated anti-rabbit antiserum (dilution 1:500). Cell nuclei were stained with DAPI (4′,6-Diamidino-2-phenylindole, SigmaAldrich) 0.5 μg/ml. Immunofluorescences were performed by triplicate and images were obtained using an Olympus FluoView FV500 confocal microscope or a Zeiss Axioskop fluorescence microscope with Nikon DS-Qi1Mc and Nikon NIS-Elements software, respectively.

### Whole-mount *In situ* hybridization

Digoxigenin-11-UTP-labeled single-stranded RNA probes for murine *Lef1, Bmp4, Msx1, Fgf9, Fgf10, Fgf4, Epfn, Dkk1, Wnt10b*, and *Shh* were prepared using a DIG RNA labeling kit (Roche Applied Science) according to the manufacturer's instructions. For whole-mount *in situ* hybridization, E11.5 branchial arches and E14.5 and E17.5 first molars cultured with 20 μM BIO or MetBIO for 24 or 48 h were fixed in 4% paraformaldehyde/PBS overnight, dehydrated in methanol, and kept at −20°C until use. Whole-mount RNA *in situ* hybridization was carried out according to Nieto et al. (1996). Some hybridized tissues were sectioned at 15 μm using a Shandom cryostome. Images were obtained with a Zeiss Stemi 2000-C stereomicroscope equipped with a Canon PowerShot A80. The number of the samples (n) for the *in situ* hybridization was 10 for each experimental condition, both control and BIO. Hybridization for each probe was performed at least twice.

### XTT-based viability assay

MDPC23 cells which derived from mouse dental pulp of between 60–70 total culture passages (Hanks et al., 1998), were cultured in standard DMEM medium (Gibco) supplemented with 20% fetal bovine serum (FBS), L-glutamine, and antibiotics. Cells were collected, counted and seeded on 96-well flat bottom plates (100 μL per well, 25000 cells/well), incubated overnight for attachment to the substrate, and then treated in triplicate with 0–100 μM MetBIO or BIO. After 24 h of treatment, XTT Cell Proliferation Assay (Roche Diagnostic) was performed following the manufacturer's protocol. Absorbance at 490 nm was measured using a spectrophotometer (Biotek Synergy HT), and MDPC-23 cells cultured in the absence of MetBIO or BIO were taken as 100% of viability.

### Statistical analysis

Statistical analyses were performed with IBM SPSS Statistics v.22 software. All data sets were subjected to a normality test prior to analysis to confirm that they followed a parametric distribution. Comparison between two groups was performed using unpaired two-tailed Student's *t*-test. Comparison between multiple groups was performed using One-way ANOVA followed by Tukey or Scheffe *post-hoc* tests. *P* ≤ 0.05 was considered to be statistically significant.

### Image analysis

Confocal and fluorescence images were analyzed using Image J (Image processing and Analysis in Java) software. Images were edited using Photoshop CS5 software.

## Results

### Overactivation of the Wnt/β-catenin pathway modifies the expression of Lef1, Msx1, Fgf8, Epfn, and Shh in the tooth initiation stage

Constitutive activation of β-catenin in the epithelial tooth buds or the deletion of β-catenin in the dental mesenchyme results in the formation of supernumerary teeth (Järvinen et al., 2006; Liu et al., 2008; Fujimori et al., 2010). To clarify the mechanism by which Wnt/β-catenin-mediated hyperdontia occurs, we studied the expression of selected genes related to the initiation of odontogenesis during the early stages of dental development, after overactivating the canonical Wnt pathway by BIO in isolated mouse branchial arches.

Previously to our study, we evaluated cell viability and apoptosis in the presence of different concentrations of BIO. We used XTT assay of dental pulp-derived MDPC-23 cells, which can be induced to differentiate into an odontoblastic phenotype under specific conditions (Hanks et al., 1998). BIO reagent induced a dose-dependent reduction of viability in MDPC-23 cells after 24 h. The EC50 value was stablished at 25 μM (Supplementary Figure 1A). When we studied the apoptotic effect of 20 μM BIO in E14.5 molars cultured for 6 days, Caspase3 was detected in cells of the oral epithelium of control and BIO-treated molars. On the contrary, BIO barely induced apoptosis in the dental epithelium and mesenchyme cells of E14.5 molars cultured for 6 days (Supplementary Figure 1B). Accordingly, concentrations of BIO between 10-20 μM were considered optimal in our organ culture experiments.

**Figure 1:**
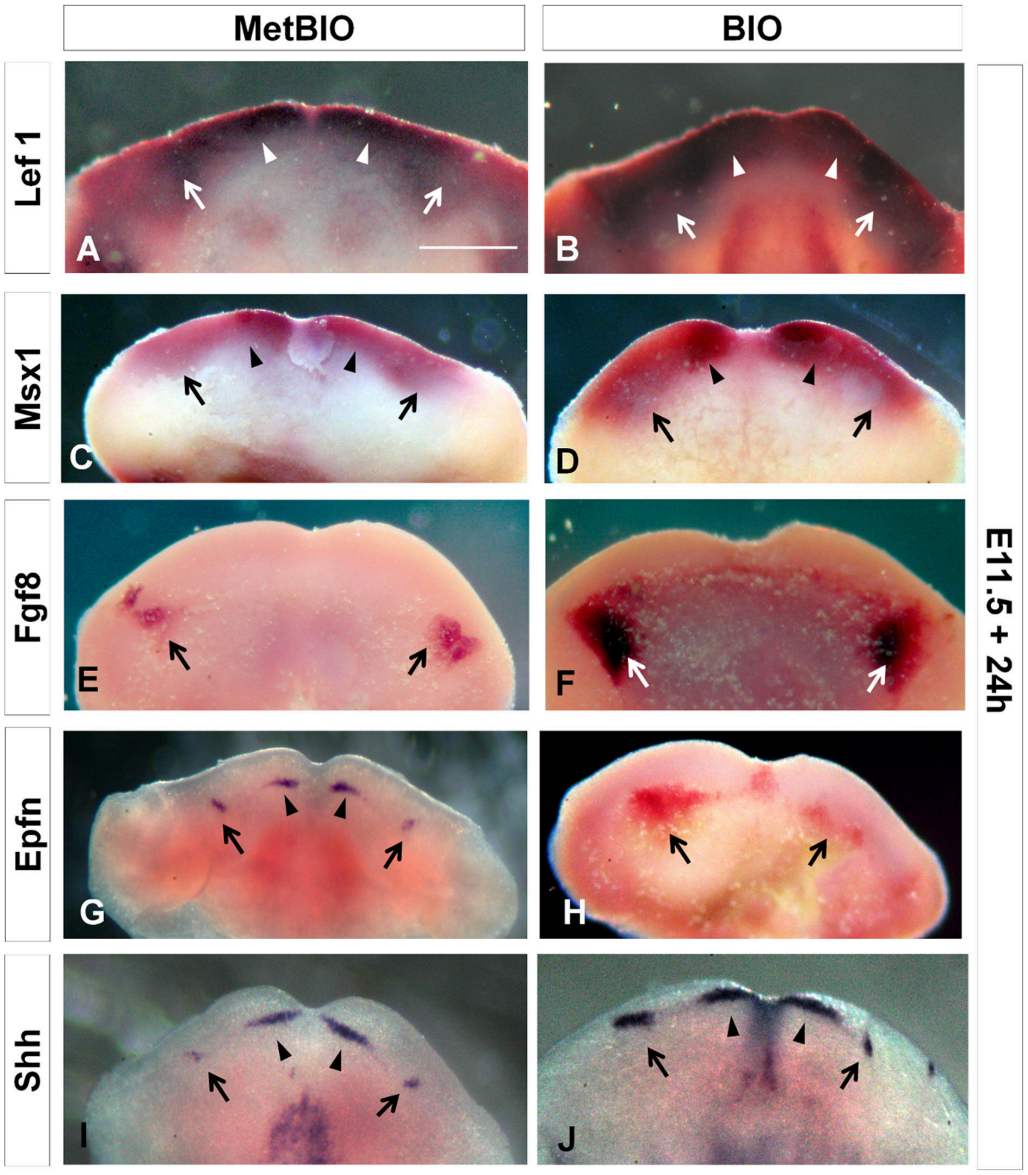
**E11.5 first branchial arches cultured with MetBIO (control) or BIO (40 μM) for 24 h**. *In situ* hybridization for *Lef1* **(A,B)**, *Msx1* **(C,D)**, *Fgf8* **(E,F)**, *Epfn* **(G,H)**, and *Shh* **(I,J)** mRNA. The expression of these genes increased and extended to the distal region of branchial arches in BIO treatment samples, when compared to controls. Arrows and arrowheads indicate the presumptive molar and incisor region, respectively. Scale bar: 200 μm.

First, we analyzed the expression of *Lef1* in E11.5 mouse lower mandibles after culture with BIO for 24 h. *Lef1* is a direct effector of Wnt/β-catenin, and increased mRNA levels for *Lef1* are hereby considered a positive control for Wnt hyperactivity. This result indicates the effectiveness of our treatment to overactivate the canonical Wnt pathway at early stages of odontogenesis (Figures 1E–J). In whole E11.5 mandibles, we found that the expression levels of *Lef1* and *Msx1* were increased after treatment with BIO in the presumptive regions of incisors and molars (Figures 1A–D). Fgf8, Epfn, and Shh are some of the major signaling molecules expressed in the oral epithelium in the initial stage of dental development. We found that in BIO-treated branchial arches, the expression levels of *Fgf8, Epfn*, and *Shh* were upregulated, and importantly, their expression fields extended ectopically to other neighboring areas (Figures 1F,H,J).

### Overactivation of the Wnt/β-catenin pathway increased alkaline phosphatase activity and odontoblast markers in the dental mesenchyme during multiple stages of tooth morphogenesis

As we described in a previous report, overactivation of the Wnt/β-catenin pathway during dental morphogenesis stage causes a delay in dental development and deficiencies in the formation of the inner dental epithelium. Under those conditions, dental mesenchymal and epithelial cells did not properly polarize and organize (Aurrekoetxea et al., 2012).

**Figure 2:**
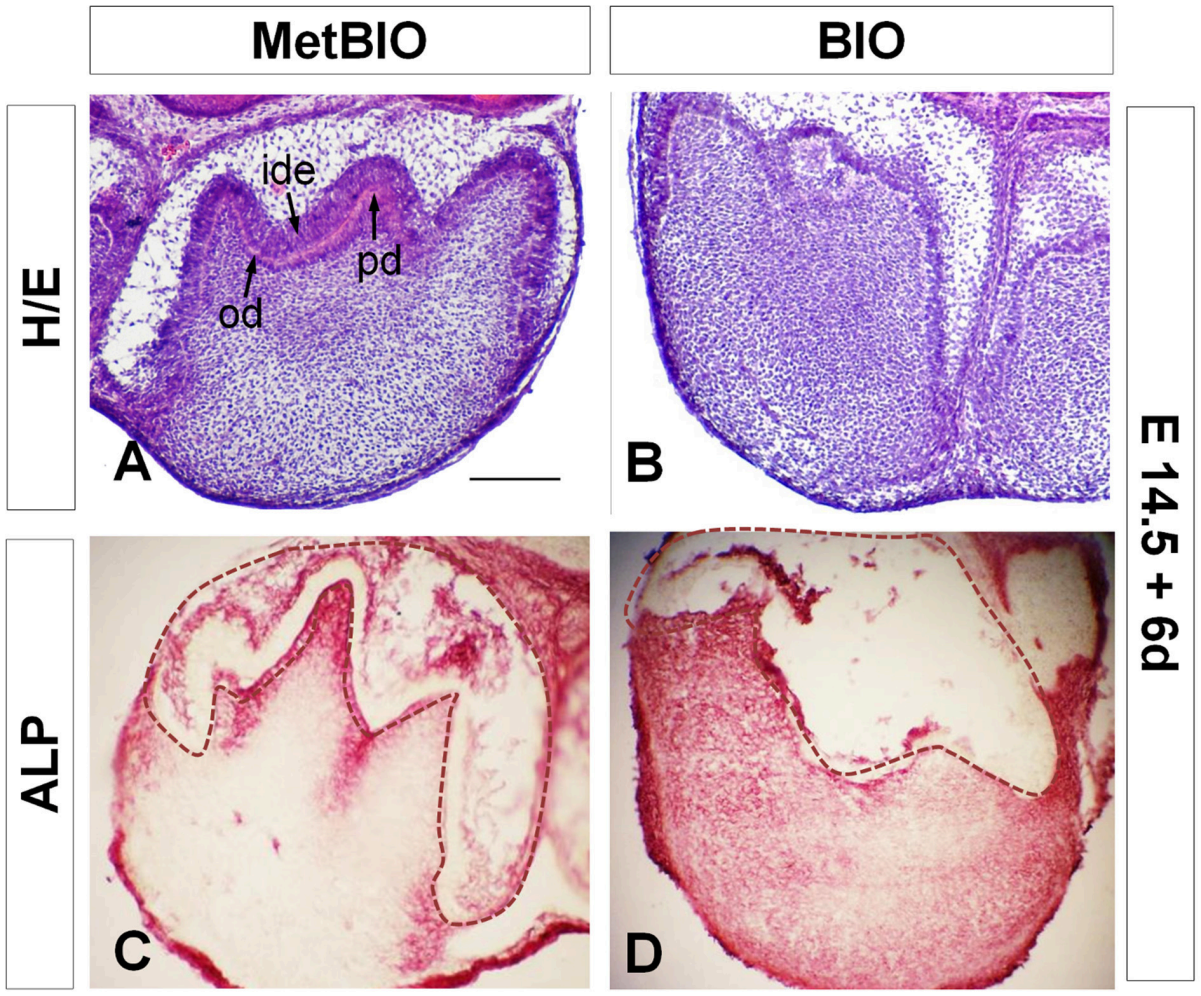
**E14.5 first molars cultured with MetBIO (control) or BIO (20 μM) for 6 days**. Control molar sections stained with hematoxylin-eosin **(A)** had normal development of dental cusps, clear polarization of the inner dental epithelia cells (ide), odontoblast differentiation (od), and a thin layer of predentin (pd). In contrast, molars cultured with BIO treatment **(B)** showed an irregular and disorganized dental cusp pattern, non-polarized cells in the inner dental epithelium and an undifferentiated odontoblastic layer. Alkaline phosphatase activity spread all over the dental mesenchyme in BIO-treated molars **(D)**, but in control samples, enzymatic activity was detected in only in the odontoblastic and subodontoblastic layers **(C)**. The dotted region corresponds to dental epithelium. Od, odontoblasts; ide, inner dental epithelium; pd, predentin. Scale bar: 200 μm.

We decided to further investigate the characteristics of these dental cells and tissue differentiation defects. Molars treated with BIO for 6 days exhibited different abnormalities, in which the inner dental epithelium was the most affected structure. In addition, dental ridges where characteristically blunted and contained no polarized epithelial cells, in contrast to the control samples, which showed polarized preameloblasts. Moreover, in BIO samples, mesenchymal odontoblastic precursor cells were completely disorganized and no dentin extracellular matrix production was detected (Figures 2A,B). As we described in a previous report, the expression of the mature odontoblast cell marker Nestin in control molars was limited to mesenchymal cells facing the inner dental epithelium. However, in BIO-treated molars, the expression of Nestin spread ectopically to subodontoblastic cell layers in the dental mesenchyme (Aurrekoetxea et al., 2012).

**Figure 3:**
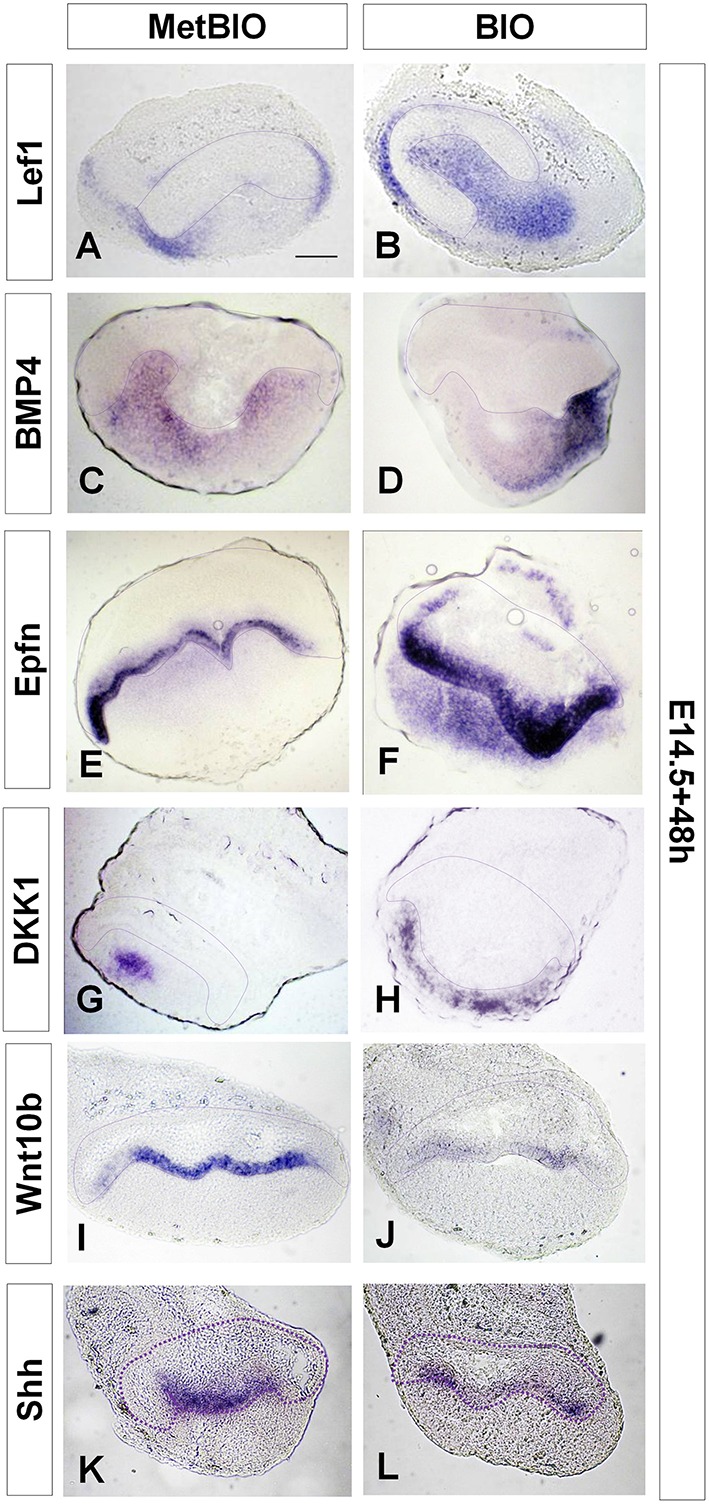
**E14.5 first molars cultured with MetBIO or BIO (20 μM) for 48 h**. *In situ* hybridization for *Lef1* **(A,B)**, *Bmp4* **(C,D)**, *Epfn* **(E,F)**, *Dkk1* **(G,H)**, *Wnt10b* **(I,J)**, and *Shh* **(K,L)**. In the BIO treatment, labeling increased for *Lef1, Dkk1, Bmp4*, and Epfn gene expression but decreased for *Wnt10b* and *Shh*. The dotted region corresponds to the enamel organ, including the inner dental epithelium. Scale bar: 200 μm.

Alkaline phosphatase (ALP) activity is an important characteristic of mineralizing cells of mesenchymal origin. Thus, we wanted to assess whether the change in Nestin expression would be paralleled by that of ALP in BIO-treated molar teeth. In control molars, ALP activity was detected in the stellate reticulum, odontoblasts, and subodontoblastic cells, whereas preameloblasts were negative for ALP activity (Figure 2C). ALP activity in BIO-treated molars completely differed from that in the controls: it was greatly increased and extended throughout the entire mesenchymal region (Figure 2D). Similarly, the increase in ALP activity in response to Wnt/β-catenin overactivation also correlated with the overexpression of other odontoblastic differentiation markers such as *Bmp4* and *Epfn* (Figure 3F) and their extension to ectopic areas in the dental papilla region.

### The Wnt/β-catenin signaling pathway controls the expression of Dkk1, Wnt10b and Shh during tooth morphogenesis

The increased expression of *Lef1*, after culture with BIO, indicates the effectiveness of our treatment to overactivate the canonical Wnt pathway at the morphogenesis stages of odontogenesis (Figures 3A,B). Similarly, Bmp4 and Epfn enhanced in dental mesenchyme of E14.5 molars cultured with BIO (Figures 3C–F).

We wanted to evaluate the effect of this sustained overactivation of Wnt/β-catenin on the expression of the natural regulators (both activators and inhibitors) of this pathway *in vivo*. *Dkk1* is expressed in the oral ectoderm at the beginning of odontogenesis (E11–12); later on, its expression is restricted to the dental mesenchyme (Fjeld et al., 2005). In our organotypic culture system, compared with the controls, BIO treatment for 48 h increased *Dkk1* expression in the dental mesenchyme of E14.5 molars (Figures 3G,H).

During *in vivo* tooth development, *Wnt10b* is expressed in the presumptive regions of incisors and molars in the first branchial arches (E11.5; Dassule and McMahon, 1998). At the stage of tooth morphogenesis (E14.5), *Wnt10b* expression is restricted to the primary enamel knot. Dkk1 downregulates *Wnt10b* expression in the primary enamel knot during tooth morphogenesis (Liu et al., 2008). In our model, overactivation of the Wnt/β-catenin pathway using BIO dramatically reduced Wnt10b expression in the inner dental epithelium during tooth morphogenesis (Figures 3I,J).

Shh is another important signaling molecule that appears to be critically associated with dental morphogenesis and dental cell differentiation (Dassule et al., 2000; Gritli-Linde et al., 2002). In our organotypic culture system, overactivation of Wnt/β-catenin induced a decrease in *Shh* expression in cultures of E14.5 molars, but the signal did not completely disappear (Figures 3K,L).

### The Wnt/β-catenin signaling pathway upregulates Fgf4 and Fgf10 expression during tooth morphogenesis

Some members of the fibroblast growth factor family, such as Fgf4 or Fgf10, are essential for cell proliferation and for the correct formation of tooth cusps (Gritli-Linde et al., 2002).

**Figure 4:**
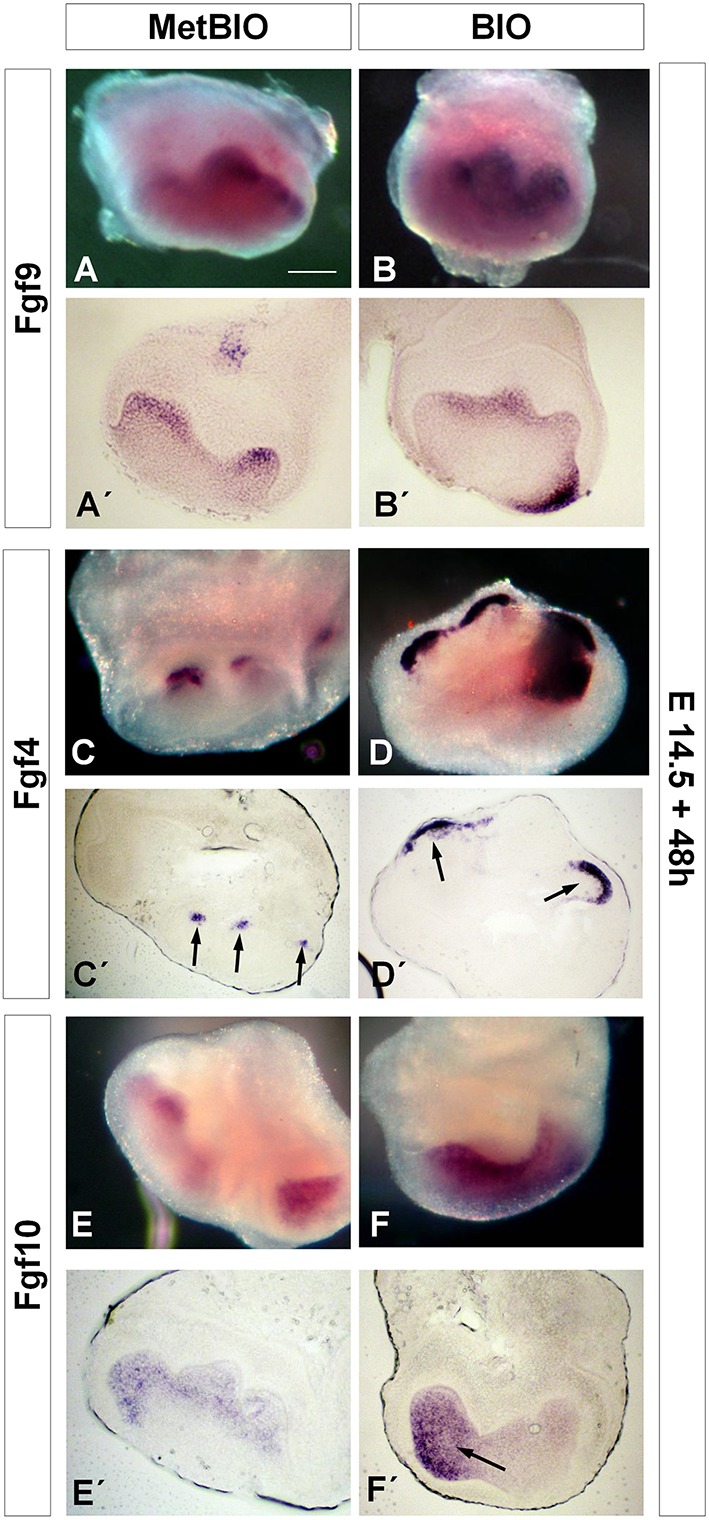
**E14.5 first molars cultured with MetBIO or BIO (20 μM) for 48 h**. *In situ* hybridization on whole molars and sections for *Fgf9* **(A,B**′**)**, *Fgf4* **(C,D**′**)**, and *Fgf10* **(E,F**′**)**. *Fgf9* was clearly expressed in the inner enamel epithelium in control cultures **(A,A**′**)**, and the staining slightly increased in BIO-treated molars **(B,B**′**)**. In control samples, *Fgf4* was expressed exclusively in the enamel knots (**C,C**′, arrows), but treatment with BIO spread the staining to the inner dental epithelium (**D,D**′, arrows). *Fgf10* labeling was restricted to the subodontoblastic layer in controls **(E,E**′**)**, but the expression spread into the dental mesenchyme in BIO cultures (**F,F**′, arrow). Scale bar: 200 μm.

We studied the expression of *Fgf* genes in molar samples after hyperactivation of Wnt/β-catenin. In our experiments, the expression of *Fgf9* increased slightly in molars treated for 48 h with BIO (Figures 4A–B′); however, the expression of *Fgf4* and *Fgf10* increased markedly (Figures 4C–F′). The most striking change in expression was observed for *Fgf4*, which was normally restricted to the enamel knots in control samples and whose presence appeared greatly enhanced and extended along the inner dental epithelium in samples treated with BIO (Figures 4D,D′). *Fgf10* expression was also upregulated, but this increase was restricted to the mesenchymal region (Figures 4E–F′).

### Overactivation of the Wnt/β-catenin signaling pathway during tooth morphogenesis and differentiation is related to an increase in mesenchymal cell proliferation and an enlargement of molar size

**Figure 5:**
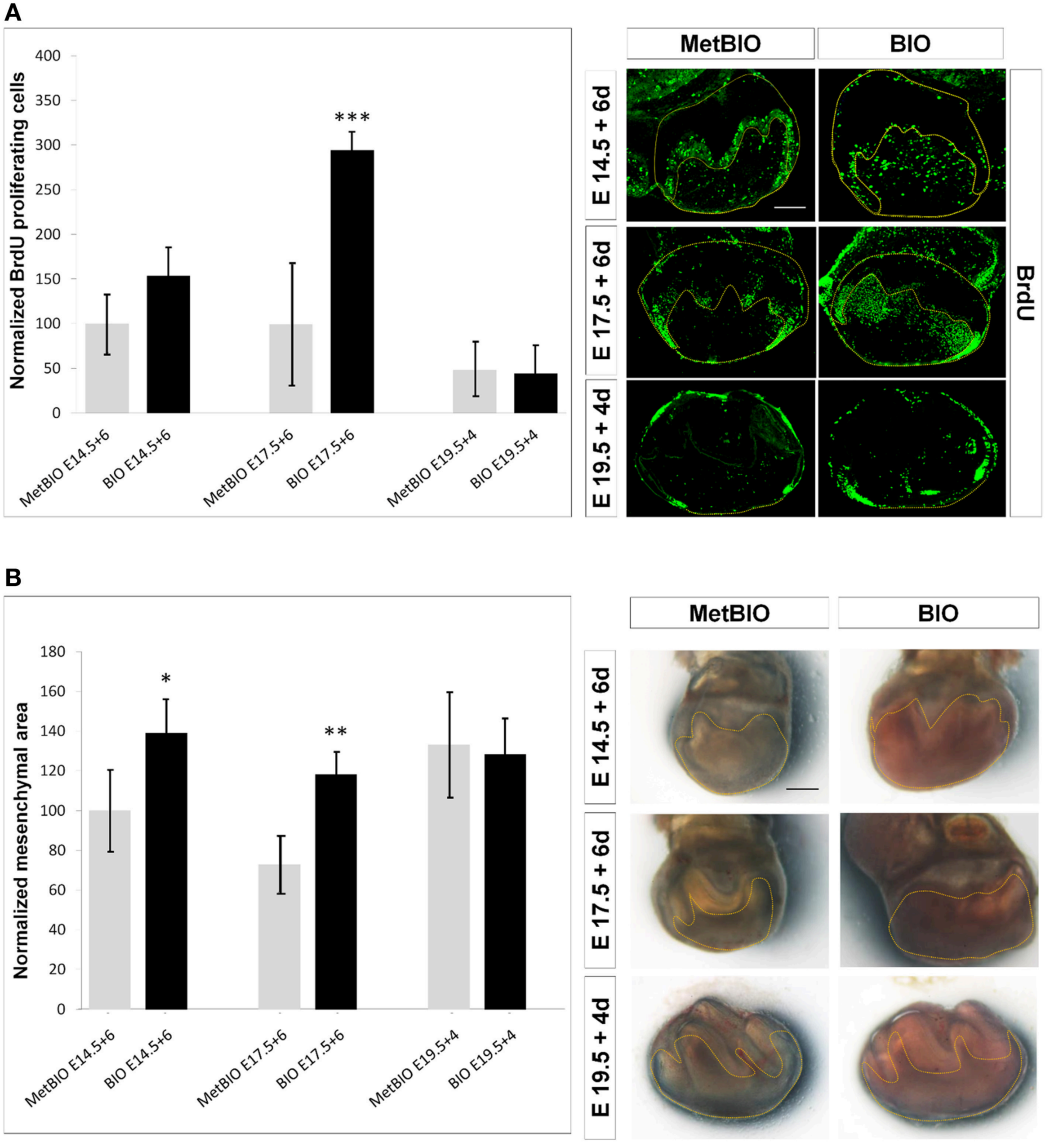
**(A)** Proliferation of dental mesenchymal cells of E14.5, E17.5, and E19.5 first molars treated with Met-BIO or BIO (20 μM) for 6 or 4 days. Graph (left) presents the normalized percentages of bromodeoxyuridine (BrdU)-positive cells from different culture samples, with respect to respective controls. Immunofluorescence for BrdU (right) of cultured molars showing positive (proliferating) cells in the dental mesenchyme. Increased cell proliferation was found in BIO-E14.5 and BIO-E17.5 (statistically significant) cultured molars. Scale bars: 200 μm. ^***^p < 0.005. **(B)** Dental mesenchyme growth of E14.5, E17.5, and E19.5 first molars treated with Met-BIO or BIO (20 μM) for 6 or 4 days. Graph (left) shows that Wnt/β-catenin overactivation promoted increased growth and enlargement of mesenchymal size when molars were treated with BIO from the tooth morphogenesis (E14.5) and predifferentiation (E17.5) stages. To the right, stereoscopic micrographs show the dental mesenchyme sizes of control and treated molars. Scale bars: 200 μm. ^*^p < 0.05; ^**^p < 0.01; ^***^p < 0.001.

Since we had observed that *Fgf* expression increased after treatment with BIO both in branchial arches and in tooth morphogenesis stages, and given the wide-known growth promoting effect of FGFs, we analyzed the rate of *in vitro* cell proliferation in first molars undergoing Wnt/β-catenin overactivation by BIO. Thus, first molars were isolated at E14.5, E17.5, and E19.5 (morphogenesis, pre-odontoblast and ameloblast cell differentiation stages, respectively) and treated with BIO for 6 or 4 days (only for E19.5 molars). First, quantification of BrdU immunohistochemical labeling revealed a significant increase in cell proliferation in the dental mesenchyme of BIO-treated E17.5 teeth compared with the control. BIO-treated E14.5 molars increased cell proliferation but not significantly, and 19.5 molars showed cell proliferation similar to that observed under control conditions (Figure 5A).

Next, we examined whether the increase in cellular proliferation was consistent with the increased size detected in E14.5 and E17.5 treated molars. Our data revealed that after treating E14.5 and 17.5 first molars with BIO for 6 days, the molar mesenchyme increased significantly in size. However, the dental papilla size of E19.5 first molars cultured with BIO for 4 days did not increase significantly (Figure 5B). These data seem to show a direct relationship between increased cell proliferation and the mesenchymal size enlargement that occurred in BIO-treated molars.

### Wnt/β-catenin signaling upregulates the expression of Epfn and Bmp4 during tooth morphogenesis and differentiation

**Figure 6:**
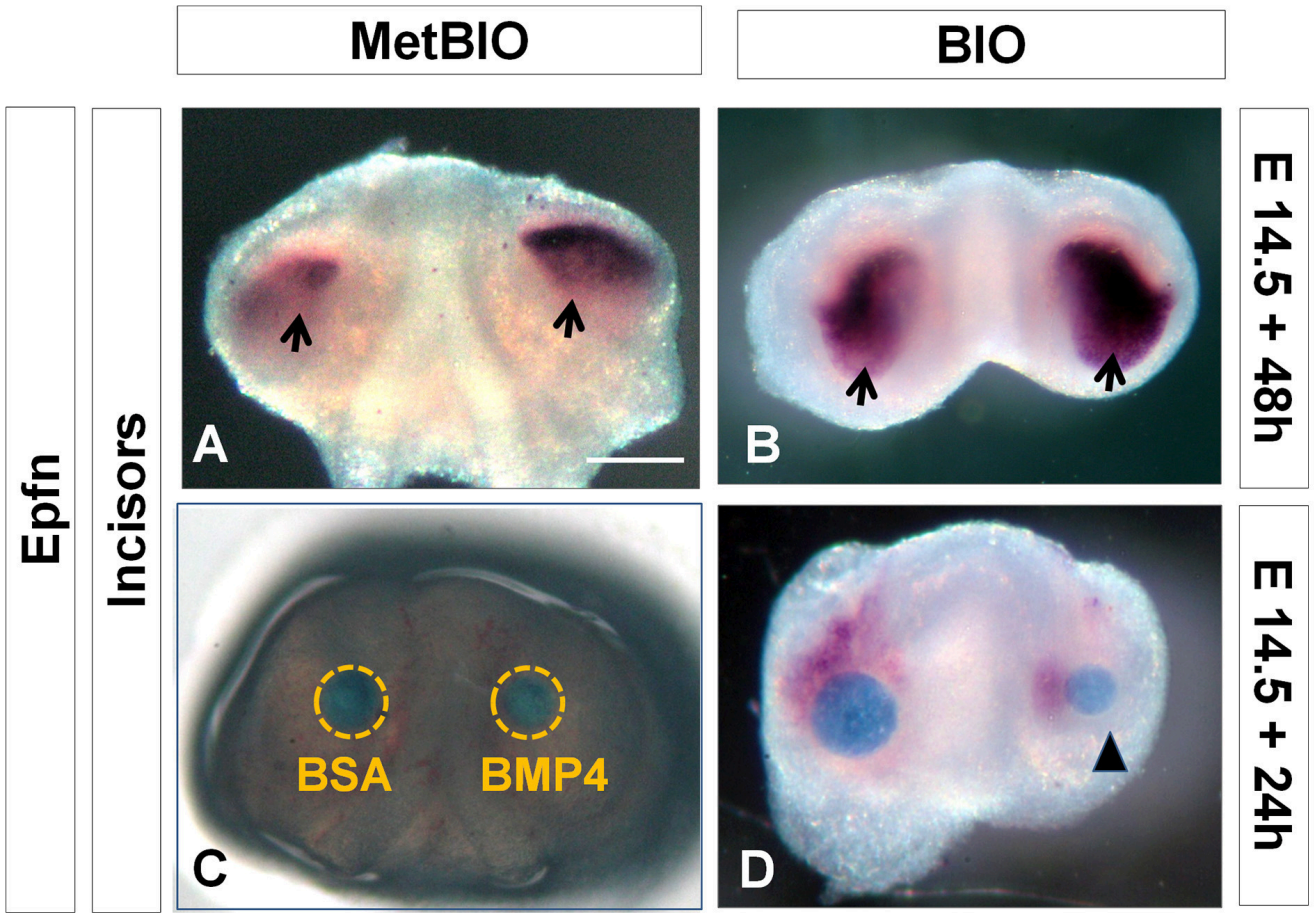
**Epfn *in situ* hybridization of E14.5 incisors cultured with microbeads containing Met-BIO, BIO (20 μM), or BMP4**. Wnt/β-catenin overactivation induced an increase in *Epfn* expression in the dental mesenchyme **(B)** compared to the control **(A)**. *In situ* hybridization for *Epfn* on E14.5 incisors cultured for 24 h in the presence of BSA (control) or Bmp4 beads **(C,D)**. Bmp4 downregulated (arrowhead) *Epfn* expression in incisors during the morphogenesis stage. Scale bar: 200 μm.

The *in situ* hybridizations performed on E14.5 tooth germs treated with BIO for 48 h revealed that *Epfn* expression extended to subodontoblastic layers of dental mesenchyme (Figure 6B), whereas in control samples, *Epfn* remained restricted to the inner dental epithelium (Figure 6A). To elucidate the overexpression relationship between *Bmp4* and *Epfn*, E14.5 incisors were cultured for 24 h in the presence of microbeads containing Bmp4 or BSA (control; Figure 6C). We observed that in the regions near the Bmp4 microbeads, the expression of *Epfn* dramatically decreased in comparison to the BSA (control; Figure 6D). These results indicated that Bmp4 downregulated *Epfn* expression in incisors, at least during the dental morphogenesis stage.

### Overactivation of the Wnt/β-catenin pathway alters morphological development and differentiation during ameloblast and odontoblast differentiation

**Figure 7:**
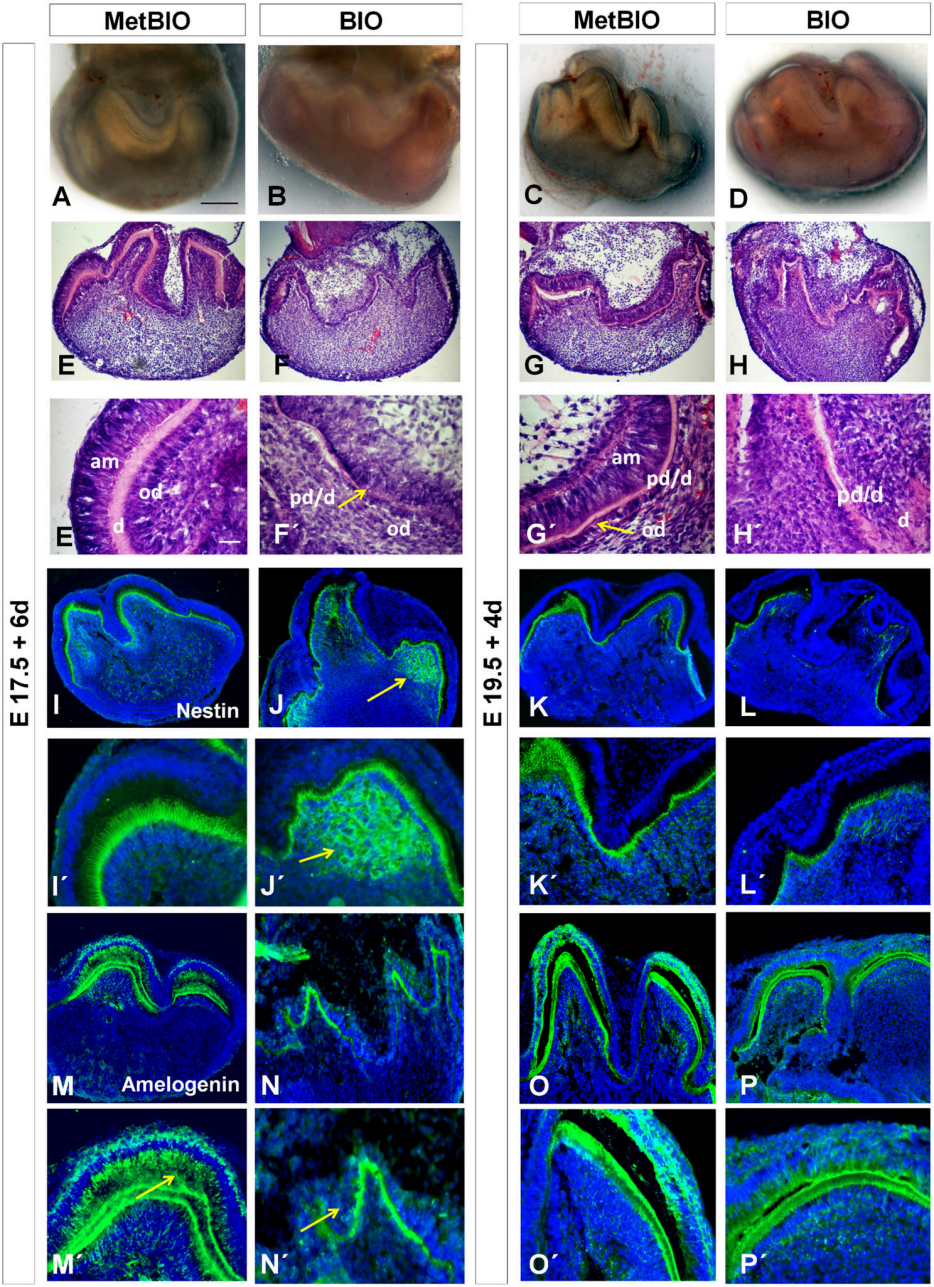
**MetBIO (control) and BIO (20 μM) treatment of E17.5 and E19.5 first molars for 6 or 4 days**. Whole molar cultures analyzed by stereoscopic microscopy **(A–D)**. Frontal sections of molars stained with hematoxylin-eosin **(E–H**′**)**. These pictures show striking differences in the growth of the inner dental epithelium and in odontoblast differentiation of control **(E,E**′**, G,G**′**)** and BIO-treated **(F,F**′**, H,H**′**)** samples. Immunofluorescence for Nestin **(I–L**′**)** and Amelogenin **(M–P**′**)** in control and BIO-treated molars. Nestin labeled odontoblasts and spread to the subodontoblastic area (arrow, **J,J**′) in E17.5 BIO-cultures, whereas in the controls **(I,I**′**)**, NNestin was restricted to the odontoblastic layer. In E19.5 cultures, both controls and treatments showed *Nestin* in odontoblasts **(K,L**′**)**. The presence of amelogenin significantly decreased **(N,N**′**)** in E17.5 BIO-treated inner dental epithelium (**N**′, arrow) compared to controls (**M,M**′, arrow). E19.5 cultures treated with BIO **(P,P**′**)** exhibited slightly reduced amelogenin expression compared to the controls **(O,O**′**)**. am, ameloblasts; od, odontoblasts; pd/d, predentin/dentin. Scale bar: 200 μm.

We studied whether the effects described in the earlier stages of tooth morphogenesis were also present at the differentiation stages of odontoblasts (E17.5) and ameloblasts (E19.5). Thus, in the presence of BIO or Met-BIO (control) we cultured E17.5 and E19.5 first molars for 6 and 4 days, respectively. In control molars, dental mesenchymal cells differentiated into functional odontoblasts secreting dentin, and the inner dental epithelial cells became well-polarized cells in the ameloblastic lineage. Moreover, these molars exhibited developed tooth cusps with predentin/dentin (Figures 7A,C,E,E′,G,G′), mesenchymal odontoblasts that stained positively for Nestin (Figures 7I,I′,K,K′), and epithelial ameloblasts that produced Amelogenin protein (Figures 7M,M′,O,O′). Additionally, first molars in E17.5 and E19.5 BIO-treated cultures showed severe developmental delays, particularly in ameloblast and odontoblast differentiation. BIO-treated molars showed an important delay in the differentiation of odontoblasts, and these cells were barely functional and produced little or no dentin at all (Figures 7B,D,F,F′,H,H′). Nestin labeling was less intense for the odontoblastic tissue area (Figures 7J,J′,L,L′) but was nevertheless extended to subodontoblastic cell layers of the dental mesenchyme, especially in E17.5 molars treated with BIO (Figures 7J,J′), similarly to our observations in E14.5 samples treated with BIO. Additionally, molars treated with BIO showed a near absence and very remarkable reduction of polarization of preameloblastic cells of the inner dental epithelium. Consistently, the presence of Amelogenin was reduced in the enamel layer of samples treated with BIO, both in cultures of E17.5 molars and in cultures of E19.5 molars (Figures 7N,N′,P,P′).

### The Wnt/β-catenin signaling pathway also controls the expression of Dkk1, Bmp4 Wnt10b, Shh and Epfn during dental differentiation

**Figure 8:**
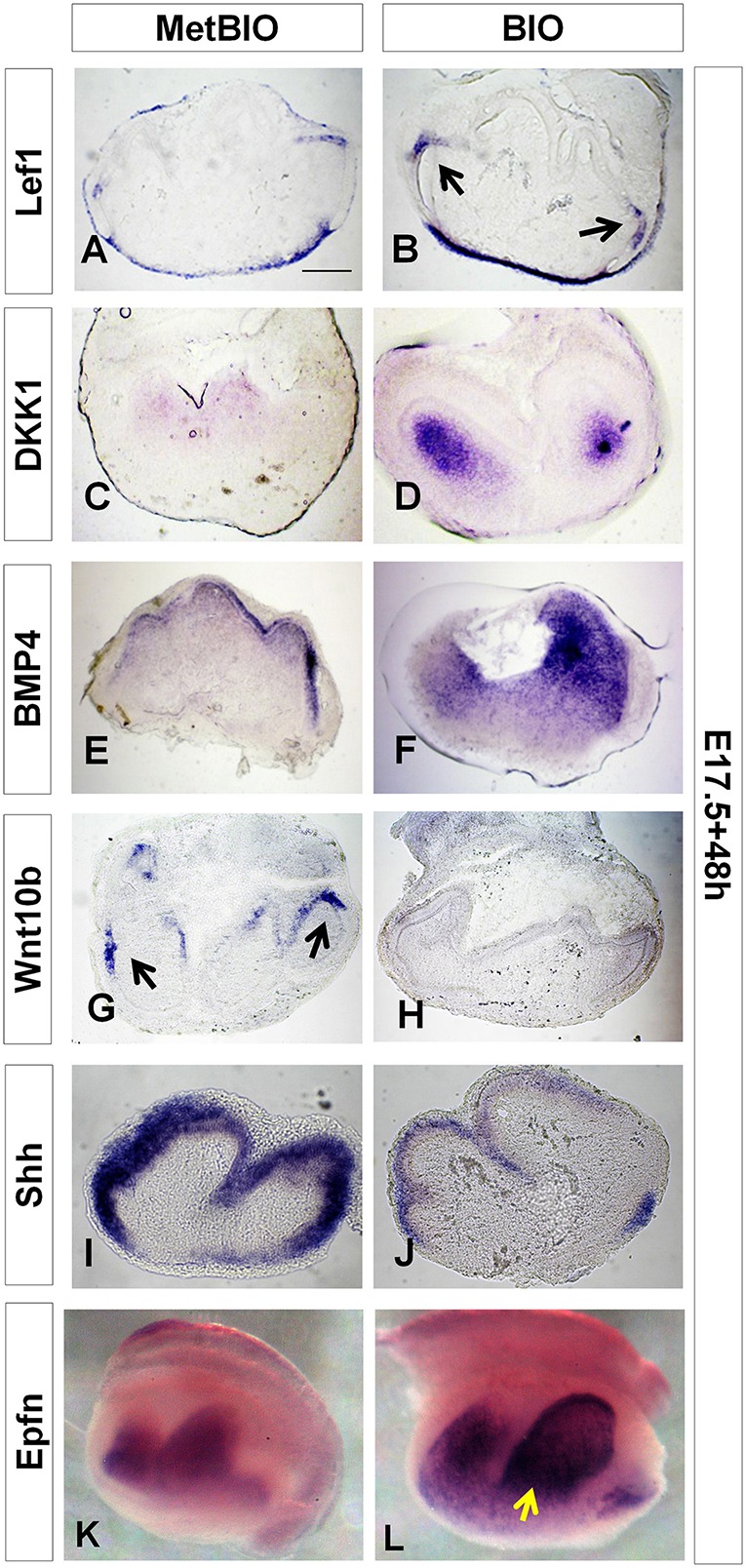
**E17.5 first molars cultured with MetBIO or BIO (20 μM) for 48 h**. *In situ* hybridization for *Lef1* **(A,B)**, *Dkk1* **(C,D)**, *Bmp4* **(E,F)**, *Wnt10b* **(G,H)**, *Shh* **(I,J)** and *Epfn* **(K,L)**. Sections of cultured molars showing that Wnt/β-catenin overactivation increased gene expression in dental epithelium (*Lef1* and *Epfn*, **B,L**) and/or dental mesenchyme (*Dkk1, Bmp4*, and *Epfn*); however, *Wnt10b* and *Shh* **(H,J)** expression was reduced with BIO treatment. Scale bars: 200 μm.

The increased expression of Lef1 after culture of E17.5 molars with BIO for 48 h indicated the effectiveness of our treatment to overactivate the canonical Wnt pathway during differentiation stages in odontogenesis (Figures 8A,B).

As occurs during the tooth morphogenesis stage, we found that during dental cell differentiation stages, the canonical Wnt signaling pathway also controlled the expression of *Dkk1, Shh, Bmp4, Wnt10b*, and *Epfn*. At E17.5, compared with the controls, expression of *Dkk1* and *Bmp4* was markedly increased in the dental mesenchyme of molars treated with BIO (Figures 8C–F). However, overactivation of Wnt/β-catenin significantly decreased the expression of *Wnt10b* and *Shh*, specifically in the inner dental epithelium of E17.5 tooth germs (Figures 8G–J). *Epfn* expression, as in the tooth morphogenesis stage, also increased in the mesenchyme of E17.5 molars after BIO treatment (Figures 8K,L).

## Discussion

In this study, we have analyzed the effects of controlled overactivation of the Wnt/β-catenin route during the different stages of odontogenesis. We treated mouse embryonic dental tissues at different developmental stages with BIO, a specific and reversible inhibitor of GSK-3 activity. GSK-3 activity *in vivo* is negatively regulated by the insulin, Wnt and reelin signaling pathways, and GSK-3 also plays a pivotal role in the Hedgehog signaling cascade. Inhibition of GSK-3 by BIO has been shown to result in the activation of the Wnt signaling pathway and in sustained pluripotency in human and mouse embryonic stem cells (ESCs; Sato et al., 2004).

Currently, there are mouse models that allow the study of constitutive activation of β-catenin in a tissue- and time-specific manner using tamoxifen-inducible Cre technology (Harada et al., 1999; Jia et al., 2013). However, in this study, we aimed to assess the effect of constitutive β-catenin activation through the inhibition of GSK-3 in the whole tooth. Due to the lack of specific Cre transgenic mouse lines that simultaneously target dental epithelium and mesenchyme, inhibition of GSK-3 in the whole dental organ with BIO was the strategy chosen for our studies. Thus, our model allowed us to analyze the specific effects of Wnt/β-catenin overactivation sequentially during the initiation (bud; E11.5), morphogenesis (cap to early bell; E14.5) and odontoblastic/ameloblastic differentiation (late bell; E17.5/E19.5) stages of odontogenesis. The results of this study stress the importance of the right balance in the activity of the Wnt/β-catenin pathway, as has been described in previous studies (Liu et al., 2008). According to this hypothesis, any anomalous activity of the pathway, by either inhibition or hyperactivation, causes structural and functional defects during odontogenesis. Moreover, in this work, we deepen the understanding of the relationship between the Wnt/β-catenin signaling pathway and the recently described transcription factor *Epfn* during odontogenesis (Nakamura et al., 2008; Jimenez-Rojo et al., 2010; Ibarretxe et al., 2012).

### Overactivation of the Wnt/β-catenin pathway promotes the formation of supernumerary teeth beginning at the initiation of odontogenesis

One important aspect of Wnt/β-catenin activity during tooth development is its involvement in the regulation of tooth number. It has been reported previously that constitutive overactivation of the Wnt/β-catenin pathway generates supernumerary teeth in genetically modified mice (Järvinen et al., 2006; Chen et al., 2009; Wang et al., 2009). The constitutive stabilization of β-catenin through epithelium-specific deletion of exon 3 of the β-catenin gene using the K14-Cre transgenic mice resulted in continuous sequential tooth production from the embryonic molar tooth germs (Järvinen et al., 2006; Liu et al., 2008). Additionally, K14-Cre-mediated epithelium-specific deletion of the *Apc* gene resulted in the formation of extra teeth next to the molar and incisor tooth germs in the *K14-Cre;Apc/cko* mutant mice (Kuraguchi et al., 2006; Wang et al., 2009). In addition, it is very intriguing that constitutive stabilization of β-catenin in the developing palatal mesenchyme causes initiation of tooth bud-like structures from the palatal epithelium (Chen et al., 2009).

In our *in vitro* model of isolated molar teeth, hyperactivation of Wnt/β-catenin during the dental morphogenesis stage (E14.5) did not generate extra teeth (Aurrekoetxea et al., 2012). This suggested that the induction of supernumerary tooth production occurred during earlier stages of dental development. For this reason, we overactivated the Wnt/β-catenin signaling pathway beginning at the tooth initiation stage. The upregulated expression of *Shh, Epfn*, and *Fgf8* in the oral epithelium after the overactivation of Wnt pathway presumably indicates the formation of supernumerary regulatory centers, which are generators of extra teeth. Therefore, the critical period for induction of supernumerary teeth by Wnt/β-catenin hyperactivation occurs exclusively at the initiation stages of odontogenesis (dental placode and dental bud), during which treatment with BIO could cause an expansion of tooth inductive potential to other neighboring oral epithelial areas. BIO exposure at the morphogenesis and differentiation stages did not cause any increase in tooth number.

### Alterations of tooth developmental patterns and dental cusps are related to changes in the expression of odontogenic target genes

Wnt/β-catenin signaling plays an important role in setting the size and morphology of the adult teeth (Sarkar and Sharpe, 2000; Cai et al., 2001). In the present study, we show that overactivation of the Wnt/β-catenin pathway during odontogenesis generates changes in dental growth and dental cell differentiation through changes in the expression patterns of certain key genes. For instance, during the transition from tooth morphogenesis to differentiation, WNT overactivation reduced *Shh* expression in the inner dental epithelium of cultured molars, whereas the expression of both *Fgf4* and *Fgf10* increased in the enamel knots and dental mesenchyme. Importantly, these changes correlated with some of the dysfunctions observed at later developmental stages, such as the impairment in ameloblast and odontoblast cell differentiation and increased dental size.

In normal tooth development, Fgf4 functions as an activator of tooth cusps, and it can partially rescue the arrest of dental development associated with the inhibition of Wnt/β-catenin signaling (Kratochwil et al., 2002). Other important downstream effectors of Wnt/β-catenin are BMPs and SHH, whose activity is perturbed under conditions of Wnt signaling manipulation (Kratochwil et al., 1996; Cobourne and Sharpe, 2010). Our data suggest that imbalances in the expression of BMPs and SHH can generate dramatic odontogenic abnormalities. Downregulation of *Shh* in the inner dental epithelium correlated with the formation of abnormally blunted dental ridges.

At the tooth morphogenesis stage, the expression of *Shh* in the enamel knot is upregulated by mesenchymal Fgf10 (Aberg et al., 2004) and downregulated by Dkk1 and Runx2 (Liu et al., 2008; Cobourne and Sharpe, 2010). In our work, the overactivation of Wnt/β-catenin increased the expression of mesenchymal *Msx1* and *Dkk1* and inhibited *Shh* expression in the dental epithelium. However, the expression of *Shh* did not disappear completely in the dental epithelium of molars treated with BIO. We propose that Epfn may play a role in this process. On the one hand, *Epfn* expression increased in dental epithelium and mesenchyme after treatment with BIO. On the other hand, in *Epfn* −/− mice, *Shh* expression is dramatically reduced in the dental preameloblastic epithelium at the bell stage (Jimenez-Rojo et al., 2010). These results indicate that expression of *Epfn* is necessary to maintain the expression of *Shh* in the epithelial region and that *Epfn* upregulation after Wnt/β-catenin overactivation prevents the complete disappearance of *Shh* from the epithelial enamel knot. Due to incomplete *Shh* signaling, dental ridges may partially form, but they will not reach the normal degree of development.

One gene that is consistently upregulated after β-catenin stabilization is *Dkk1*. This is consistent with a feedback inhibitory effect to maintain Wnt/β-catenin activity within a physiological range. This feedback switch appears to be active both during dental morphogenesis (E14.5) and during dental differentiation (E17.5), based on the responses to stimulation with BIO. These findings reinforce the view that precise control of the activity of the Wnt/β-catenin pathway is necessary for proper tooth development. Therefore, isolated cultured molars react to BIO treatment by switching off canonical Wnt activity in an attempt to counterbalance this pharmacologically induced hyperactivity.

### Deregulation of Epfn, ALP, and nestin expression suggests the onset of ectopic odontoblast-like differentiation in BIO-treated molars

Previous results have shown an inductive relationship of *Msx1* and *Bmp4* in the dental mesenchyme during the transition from initiation to morphogenesis stage of odontogenesis (Bei and Maas, 1998; Kong et al., 2011). Likewise, it has also been shown that the Wnt/β-catenin pathway promotes the expression of *Msx1, Bmp4*, and *Fgf4* (Cobourne and Sharpe, 2010; Galluccio et al., 2012).

After β-catenin stabilization by BIO exposure, *Epfn* expression increased in the dental mesenchyme in the same way that Nestin did. Epfn protein is a transcription factor essential for proper odontoblast differentiation (Nakamura et al., 2008). In molars treated with BIO, odontoblast differentiation was delayed, which was also demonstrated by a reduction of Nestin immunolabeling and alkaline phosphatase activity, specifically in the areas containing the presumptive odontoblastic cells. However, the expression of Nestin and *Epfn* was largely increased in other areas of the dental papilla, which seems to be related to an ectopic mesenchymal differentiation to odontoblast-like cells. In addition, ALP labeling was much higher in BIO-treated dental mesenchyme than in control mesenchyme, again indicating a mesenchymal ectopic mineralization. Results from other groups showed that a similar phenotype is present in genetically modified mice overexpressing β-catenin in the dental mesenchyme (Chen et al., 2009; Kim et al., 2011). The occurrence of ectopic dentin and odontoblast differentiation in the dental pulp is associated with a human pathology called intrapulpal calcifications. This disease is characterized by the presence of large numbers of pulp stones in the dental mesenchyme. The presence of these pulp stones is very common in human teeth. Several factors that may cause this disease have been already described. However, it is known that there are also other stimuli that cause this disease and that have not yet been described. Moreover, some authors associate these pulp stones with regressive or degenerative changes (Zmener, 2009). This disease would be related to the activation of mechanisms governing odontoblast cell differentiation, such as the Wnt pathway. Therefore, overactivation of canonical Wnt signaling and the correlated changes in the expression of *Bmp4*, Nestin and mesenchymal *Epfn* may be a causative factor of this condition.

### Overactivation of Wnt/β-catenin increases mesenchymal cell proliferation at different stages of odontogenesis, causing increased dental size

The relationship between the Wnt/β-catenin pathway and cell proliferation has been studied in previous works, but the reported data depended on the analyzed tissue, and in some cases, the results were contradictory (Siriwardena et al., 2009; Manceur et al., 2011). In our previous work, we found that stabilization of β-catenin during the tooth morphogenesis stage stimulated cell survival and renewal in the mesenchymal region of the tooth (Aurrekoetxea et al., 2012). In the present manuscript, we show how this increase in cell proliferation is not exclusive to the tooth morphogenesis stage and that it also occurs during the stages of odontoblast and ameloblast differentiation. As a consequence of this effect on cell proliferation, the volume of dental mesenchyme significantly increased in BIO-treated molar teeth.

Other studies have linked the expression of *Fgf4* and *Fgf8* with mesenchymal proliferation and delayed dental development (Kettunen et al., 2000; Gritli-Linde et al., 2002). Based on these data, we suggest that the significantly increased mesenchymal proliferation in the molars treated with BIO in different stages of odontogenesis is related to the dramatic increase in epithelial *Fgf4* and *Fgf10* expression detected in these molars. In summary, we can conclude that the stabilization of nuclear β-catenin induces increased FGF production and that this may be the reason for the significant increase in dental mesenchyme proliferation.

### Wnt/β-catenin signaling induces both BMP and Epfn expression, and BMP regulates Epfn through a negative feedback loop

Canonical Wnt/β-catenin pathway activity in the dental mesenchyme is closely related to the production of BMPs, and this induction controls multiple aspects of dental development, such as dental morphogenesis, the number of teeth, and dental cell differentiation (Shu et al., 2005; Hill et al., 2006; Chen et al., 2009; Fujimori et al., 2010). Our new data confirm that there is a positive inductive relationship between Wnt/β-catenin and BMP (Wnt-BMP) and that this induction is maintained during both dental morphogenesis (E14.5) and dental differentiation (E17.5).

**Figure 9:**
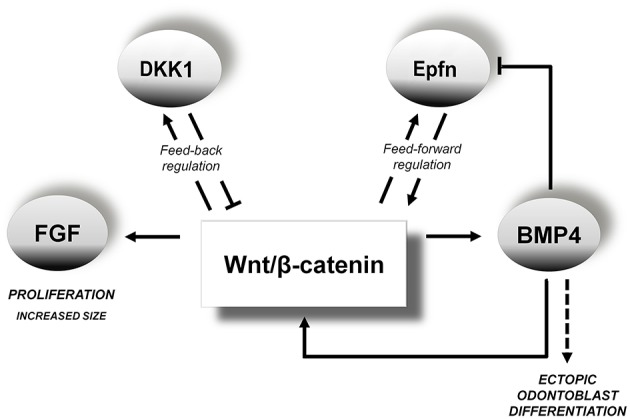
**Proposed model for the role of the Wnt/β-catenin pathway during odontogenesis**. Overactivation of the Wnt/β-catenin pathway induces increased FGF expression. This may explain the significant increase in dental mesenchyme proliferation that causes an increase in tooth size. Based on our data showing increased *Dkk1* expression after activation of the Wnt pathway, we propose a feedback loop between the Wnt/β-catenin signaling pathway and *Dkk1*. Our data confirm that there is a positive inductive relationship between Wnt/β-catenin and BMP. Overactivation of canonical Wnt signaling causes activation of *Bmp4*, Nestin and *Epfn* in the dental mesenchyme, which suggests ectopic differentiation of odontoblast-like cells. Based on our results, we propose a positive regulation between Wnt/β-catenin and *Epfn*. Our present results suggest the presence of a positive feedback loop between *Epfn* and Wnt/β-catenin. Similarly, our microbead results indicate the existence of a negative feedback loop between *Epfn* and *Bmp4*.

To date, no conclusive data had been obtained to explain in detail the relationship between *Epfn* and Wnt-BMP during dental development. We first reported the striking dental phenotype of *Epfn*-null mice (Nakamura et al., 2008). Later, we found that the absence of *Epfn* caused a decrease in the expression of *Bmp4* and *Dkk1* in early branchial arches (Jimenez-Rojo et al., 2010), which initially suggested a positive regulation between *Epfn* and BMP in the Wnt/β-catenin pathway. Subsequently, we showed that *Epfn* transfection of dental papilla-derived MDPC-23 cells induced a nuclear and cytoplasmic increase in β-catenin (Ibarretxe et al., 2012). Finally, the present results on dental germs induced to hyperactivate Wnt/β-catenin revealed a significant increase in *Epfn* expression in the dental epithelium and the dental mesenchyme. The data presented here suggest the presence of a positive feedback loop between *Epfn* and β-catenin, in which each activates the other, with the balance of their expression being essential for proper tooth development (Figure 9). In view of our results, we propose a positive regulation between Wnt/β-catenin and Epfn, which would be fundamental to determine the development and differentiation of the inner dental epithelium toward ameloblasts and of dental mesenchymal cells toward odontoblasts.

Our results with microbeads showed that local application of Bmp4 downregulated *Epfn* expression during the dental morphogenesis stage. These data indicate the existence of a negative feedback loop between Wnt/β-catenin, *Epfn* and *Bmp4* during tooth morphogenesis. According to this model, Wnt/β-catenin would activate BMP and *Epfn*, but the levels of expression of *Epfn* would be kept tightly regulated by BMP through a negative feedback mechanism. These novel findings may have important implications for our understanding of the regulation of tooth development. Thus, we propose that during dental development, *Epfn* expression would be highly restricted by the coordinated action of Wnt-BMP signaling, and this would govern fundamental processes such as tooth morphogenesis and ameloblast/odontoblast differentiation, as we first found by Nakamura et al. (2008). Our future works are designed to depeen the role of Epfn in the differentiation of ameloblasts and odontoblasts.

Finally, our studies reveal that inhibition of GSK-3 with BIO disrupts normal tooth development. GSK-3 has been proposed as a therapeutic target for several human diseases. GSK-3 inhibitors, particularly small-molecule inhibitors, are currently being considered for the treatment of such pathologies (Phukan et al., 2010). Indirubin-3′-oxime (BIO) is a potent inhibitor of GSK-3, and it has been reported to inhibit cell proliferation and arrest cell cycle progression of various cancer cells. The therapeutic use of these compounds in combating proliferative diseases such as cancer, restenosis, and psoriasis has been proposed (Cuong et al., 2010). Regarding diseases affecting dental tissues, inhibition of GSK-3 has been shown to abolish the bone loss that occurs in periodontal disease in mice (Adamowicz et al., 2012). These findings suggest the potential use of small molecules such as BIO to treat dental diseases. Thus, understanding the effects of BIO in teeth is essential to avoid undesirable side effects that may affect normal dental function.

## Author contributions

MA: Concept and design of the research, experimental work, data analysis and interpretation and writing of the article. II: Experimental work. PG: Experimental work. LJ: Concept and design of the research. TN: Concept and design of the research. YY: Concept and design of the research. GI: Concept and design of the research, data analysis and interpretation. FU: Concept and design of the research, data analysis and interpretation, writing of the article.

## Funding

This work was financed with grants from the University of the Basque Country (UFI/11-44) and the Basque Government (IT831-13). MA and PG received a fellowship from the University of the Basque Country.

### Conflict of interest statement

The authors declare that the research was conducted in the absence of any commercial or financial relationships that could be construed as a potential conflict of interest.

## Supplementary material

Supplementary Figure 1 **Cell viabilty and apoptosis analysis. (A)** XTT assay for cell viability was performed in MDPC-23 cells cultured with 0-100 μM MetBIO or BIO for 24 h. Effective concentration EC50 was determined at 25 μM BIO. **(B)** Immunofluorescence for Caspase3 in E14.5 molars cultured for 6 days in the presence of 20 μM MetBIO or BIO. Apoptotic cells were mainly detected in the oral epithelium. Only a few apoptosis appeared in the enamel organ of the tooth rudiments, both in control and treated samples. Red dots were drawn to show the frontier between dental epithelium and mesenchyme. Scale bars: 200 μm.

## Abbreviations

ALP: Alkaline phosphatase
BIO: 6-bromoindirubin-3′-oxime
BrdU: 5-bromo-2′-deoxyuridine
Epfn: Epiprofin/Sp6
E14.5, E17.5, E19.5, embryonic day 14.5, 17.4: 19.5
GSK-3: glycogen synthase kinase 3
MetBIO: methyl-BIO
Wnt: Wingless integration site.

## References

[B1] Aberg T. Wang X. P. Kim J. H. Yamashiro T. Bei M. Rice R.. (2004). Runx2 mediates FGF signaling from epithelium to mesenchyme during tooth morphogenesis. Dev. Biol. 270, 76–93. 10.1016/j.ydbio.2004.02.012 15136142

[B2] Adaimy L. Chouery E. Megarbane H. Mroueh S. Delague V. Nicolas E.. (2007). Mutation in WNT10A is associated with an autosomal recessive ectodermal dysplasia: the odonto-onycho-dermal dysplasia. Am. J. Hum. Genet. 81, 821–828. 10.1086/520064 17847007 PMC1973944

[B3] Adamowicz K. Wang H. Jotwani R. Zeller I. Potempa J. Scott D. A. (2012). Inhibition of GSK3 abolishes bacterial-induced periodontal bone loss in mice. Mol. Med. 18, 1190–1196. 10.2119/molmed.2012.00180 22847803 PMC3510296

[B4] Ahn Y. Sanderson B. W. Klein O. D. Krumlauf R. (2010). Inhibition of Wnt signaling by Wise (Sostdc1) and negative feedback from Shh controls tooth number and patterning. Dev. 137, 3221–3231. 10.1242/dev.054668 20724449 PMC6512258

[B5] Andl T. Reddy S. T. Gaddapara T. Millar S. E. (2002). WNT signals are required for the initiation of hair follicle development. Dev. Cell. 2, 643–653. 10.1016/S1534-5807(02)00167-3 12015971

[B6] Aurrekoetxea M. Lopez J. García P. Ibarretxe G. Unda F. (2012). Enhanced Wnt/β-catenin signalling during tooth morphogenesis impedes cell differentiation and leads to alterations in the structure and mineralisation of the adult tooth. Biol. Cell. 104, 603–617. 10.1111/boc.201100075 22671936

[B7] Barrientos S. Stojadinovic O. Golinko M. S. Brem H. Tomic-Canic M. (2008). Growth factors and cytokines in wound healing. Wound Repair Regen. 16, 585–601. 10.1111/j.1524-475X.2008.00410.x 19128254

[B8] Bei M. Maas R. (1998). FGFs and BMP4 induce both Msx-1-independent and Msx1-dependent signalling pathways in early tooth development. Development 125, 4325–4433. 9753686 10.1242/dev.125.21.4325

[B9] Cai Y. Lechner M. S. Nihalani D. Prindle M. J. Holzman L. B. Dressler G. R. (2001). Phosphorylation of Pax2 by the c-Jun N-terminal kinase and enhanced Pax2-dependent transcription activation. J. Biol. Chem. 277, 1217–1222. 10.1074/jbc.M109663200 11700324

[B10] Chen J. Lan Y. Baek J. A. Gao Y. Jiang R. (2009). Wnt/beta-catenin signaling plays an essential role in activation of odontogenic mesenchyme during early tooth development. Dev. Biol. 334, 174–185. 10.1016/j.ydbio.2009.07.015 19631205 PMC2752344

[B11] Cobourne M. T. Sharpe P. T. (2010). Making up the numbers: the molecular control of mammalian dental formula. Semin. Cell Dev. Biol. 21, 314–324. 10.1016/j.semcdb.2010.01.007 20080198

[B12] Cuong N. M. Tai B. H. Hoan D. H. Huong T. T. Kim Y. H. Hyun J. H.. (2010). Inhibitory effects of indirubin derivatives on the growth of HL-60 leukemia cells. Nat. Prod. Commun. 5, 103–106. 20184032

[B13] D'Andrea L. D. Del Gatto A. De Rosa L. Romanelli A. Pedone C. (2009). Peptides targeting angiogenesis related growth factor receptors. Curr. Pharm. Des. 15, 2414–2429. 10.2174/138161209788682235 19601840

[B14] Dassule H. R. McMahon A. P. (1998). Analysis of epithelial.mesenchymal interactions in the initial morphogenesis of the mammailan tooth. Dev. Biol. 202, 215–227. 10.1006/dbio.1998.8992 9769173

[B15] Dassule H. R. Lewis P. Bei M. Maas R. McMahon A. P. (2000). Sonic Hedgehog regulates growth and morphogenesis of the tooth. Development 127, 4775–4785. 11044393 10.1242/dev.127.22.4775

[B16] Fjeld K. Kettunen P. Furmanek T. Kvinnsland I. H. Luukko K. (2005). Dynamic expression of Wnt signaling-related Dickkopf1, -2, and -3 mRNAs in the developing mouse tooth. Dev. Dyn. 233, 161–166. 10.1002/dvdy.20285 15759274

[B17] Fujimori S. Novak H. Weissenböck M. Jussila M. Gonçalves A. Zeller R.. (2010). Wnt/β-catenin signaling in the dental mesenchyme regulates incisor development by regulating Bmp4. Dev. Biol. 348, 97–106. 10.1016/j.ydbio.2010.09.009 20883686 PMC2997430

[B18] Galluccio G. Castellano M. La Monaca C. (2012). Genetic basis of non-syndromic anomalies of human tooth number. Arch. Oral Biol. 57, 918–930. 10.1016/j.archoralbio.2012.01.005 22325622

[B19] Gritli-Linde A. Bei M. Maas R. Zhang X. M. Linde A. McMahon A. P. (2002). Shh signalling within the dental epithelium is necessary for cell proliferation, growth and polarization. Development 129, 5323–5337. 10.1242/dev.00100 12403705

[B20] Hanks C. T. Fang D. Sun Z. Edwards C. A. Butler W. T. (1998). Dentin specific proteins in MDPC-23 cell line. Eur. J. Oral Sci. 106, 260–266. 10.1111/j.1600-0722.1998.tb02185.x 9541235

[B21] Hill T. P. Taketo M. M. Birchmeier W. Hartmann C. (2006). Multiple roles of mesenchymal beta-catenin during murine limb patterning. Development 133, 1219–1229. 10.1242/dev.02298 16495310

[B22] Ibarretxe G. Aurrekoetxea M. Crende O. Badiola I. Jimenez-Rojo L. Nakamura T.. (2012). Epiprofin/Sp6 regulates Wnt-BMP signaling and the establishment of cellular junctions during the bell stage of tooth development. Cell Tissue Res. 350, 95–107. 10.1007/s00441-012-1459-8 22868911

[B23] Harada N. Tamai Y. Ishikawa T. Sauer B. Takaku K. Oshima M.. (1999). Intestinal polyposis in mice with a dominant stable mutation of the beta-catenin gene. EMBO J. 18, 5931–5942. 10.1093/emboj/18.21.5931 10545105 PMC1171659

[B24] Järvinen E. Salazar-Ciudad I. Birchmeier W. Taketo M. M. Jernvall J. Thesleff I. (2006). Continuous tooth generation in mouse is induced by activated epithelial Wnt/beta-catenin signaling. Proc. Natl. Acad. Sci. U.S.A. 103, 18627–18632. 10.1073/pnas.0607289103 17121988 PMC1693713

[B25] Jernvall J. Thesleff I. (2000). Reiterative signaling and patterning during mammalian tooth morphogenesis. Mech. Dev. 92, 19–29. 10.1016/S0925-4773(99)00322-6 10704885

[B26] Jia M. Chen S. Zhang B. Liang H. Feng J. Zong Z. (2013). Effects of constitutive beta-catenin activation on vertebral bone growth and remodeling at different postnatal stages in mice. PLoS ONE 8:e74093. 10.1371/journal.pone.0074093 24066100 PMC3774640

[B27] Jiang N. Zhou J. Chen M. Schiff M. D. Lee C. H. Kong K.. (2013). Postnatal epithelium and mesenchyme stem/progenitor cells in bioengineered amelogenesis and dentinogenesis. Biomaterials 35, 2172–2180. 10.1016/j.biomaterials.2013.11.061 24345734 PMC4112409

[B28] Jimenez-Rojo L. Ibarretxe G. Aurrekoetxea M. de Vega S. Nakamura T. Yamada Y.. (2010). Epiprofin/Sp6: A new player in the regulation of tooth development. Histol Histopathol. 25, 1621–1630. 20886441 10.14670/HH-25.1621

[B29] Kantaputra P. Sripathomsawat W. (2011). WNT10A and isolated hypodontia. Am. J. Med. Genet. A 155A, 1119–1122. 10.1002/ajmg.a.33840 21484994

[B30] Kettunen P. Laurikkala J. Itäranta P. Vainio S. Itoh N. Thesleff I. (2000). Associations of FGF-3 and FGF-10 with signaling networks regulating tooth morphogenesis. Dev. Dyn. 219, 322–332. 11066089 10.1002/1097-0177(2000)9999:9999<::AID-DVDY1062>3.0.CO;2-J

[B31] Kim T. H. Lee J. Baek J. Lee J. C. Yang X. Taketo M. (2011). Constitutive stabilization of β-catenin in the dental mesenchyme leads to excessive dentin and cementum formation. Biochem. Biophys. Res. Commun. 412, 549–555. 10.1016/j.bbrc.2011.07.116 21854758

[B32] Kong H. Wang Y. Patel M. Mues G. D'souza R. N. (2011). Regulation of bmp4 expression in odontogenic mesenchyme: from simple to complex. Cells Tissues Organs (Print). 194, 156–160. 10.1159/000324747 21546760 PMC3178073

[B33] Kratochwil K. Dull M. Fariñas I. Galceran J. Grosschedl R. (1996). Lef1 expression is activated by BMP-4 and regulates inductive tissue interactions in tooth and hair development. Genes Dev. 10, 1382–1394. 8647435 10.1101/gad.10.11.1382

[B34] Kratochwil K. Galceran J. Tontsch S. Roth W. Grosschedl R. (2002). FGF4, a direct target of LEF1 and Wnt signaling, can rescue the arrest of tooth organogenesis in Lef1(-/-) mice. Genes Dev. 16, 3173–3185. 10.1101/gad.1035602 12502739 PMC187508

[B35] Kuraguchi M. Wang X. P. Bronson R. T. Rothenberg R. Ohene-Baah N. Y. Lund J. J.. (2006). Adenomatous polyposis coli (APC) is required for normal development of skin and thymus. PLoS Genet. 2:e146. 10.1371/journal.pgen.0020146 17002498 PMC1564426

[B36] Liu F. Chu E. Y. Watt B. Zhang Y. Gallant N. M. Andl T.. (2008). Wnt/beta-catenin signaling directs multiple stages of tooth morphogenesis. Dev. Biol. 313, 210–224. 10.1016/j.ydbio.2007.10.016 18022614 PMC2843623

[B37] Liu F. Millar S. E. (2010). Wnt/beta-catenin signaling in oral tissue development and disease. J. Dent. Res. 89, 318–330. 10.1177/0022034510363373 20200414 PMC3140915

[B38] Logan C. Y. Nusse R. (2004). The Wnt signaling pathway in development and disease. Annu. Rev. Cell Dev. Biol. 20, 781–810. 10.1146/annurev.cellbio.20.010403.113126 15473860

[B39] Lohi M. Turker A. S. Sharpe P. (2010). Expression of Axin2 indicates a role for canonical Wnt signaling in development of the crown and root during pre- and postnatal tooth development. Dev. Dyn. 239, 160–167. 19653310 10.1002/dvdy.22047

[B40] Lumsden A. G. (1988). Spatial organization of the epithelium and the role of neural crest cells in the initiation of the mammailan tooth germ. Development 103, 155–169. 3250849 10.1242/dev.103.Supplement.155

[B41] Manceur A. P. Tseng M. Holowacz T. Witterick I. Weksberg R. McCurdy R. D.. (2011). Inhibition of glycogen synthase kinase-3 enhances the differentiation and reduces the proliferation of adult human olfactory epithelium neural precursors. Exp. Cell Res. 317, 2086–2098. 10.1016/j.yexcr.2011.06.004 21708147

[B42] Martin A. Unda F. J. Begue-Kirn C. Ruch J. V. Arechaga J. (1998). Effects of aFGF, bFGF, TGFbeta 1 and IGF-I on odontoblast differentiation *in vitro*. Eur. J. Oranl Sci. 106, 117–121. 10.1111/j.1600-0722.1998.tb02162.x 9541212

[B43] Massink M. P. Créton M. A. Spanevello F. Fennis W. M. Cune M. S. Savelberg S. M.. (2015). Loss-of-function mutations in the WNT co-receptor LRP6 cause autosomal-dominant oligodontia. Am. J. Hum. Genet. 97, 621–626. 10.1016/j.ajhg.2015.08.014 26387593 PMC4596913

[B44] Meijer L. Skaltsounis A. L. Magiatis P. Polychronopoulos P. Knockaert M. Leost M.. (2003). GSK-3-selective inhibitors derived from Tyrian purple indirubins. Chem. Biol. 10, 1255–1266. 10.1016/j.chembiol.2003.11.010 14700633

[B45] Mina M. Collar E. J. (1987). The induction of odontogenesis in non-dental mesenchyme combined with early murine mandibular arch epithelium. Arch. Oral Biol. 32, 123–127. 10.1016/0003-9969(87)90055-0 3478009

[B46] Muto T. Miyoshi K. Horiguchi T. Hagita H. Noma T. (2012). Novel genetic linkage of rat Sp6 mutation to Amelogenesis imperfecta. Orphanet J. Rare Dis. 7:34. 10.1186/1750-1172-7-34 22676574 PMC3464675

[B47] Nakamura T. Unda F. de-Vega S. Vilaza A. Fukumoto S. Yamada K. M.. (2004). The Krüppel-like factor epiprofin is expressed by epithelium of developing teeth, hair follicles, and limb buds and promotes cell proliferation. J. Biol. Chem. 279, 626–634. 10.1074/jbc.M307502200 14551215

[B48] Nakamura T. de Vega S. Fukumoto S. Jimenez L. Unda F. Yamada Y. (2008). Transcription factor epiprofin is essential for tooth morphogenesis by regulating epithelial cell fate and tooth number. J. Biol. Chem. 283, 4825–4833. 10.1074/jbc.M708388200 18156176

[B49] Nakamura T. Yoshitomi Y. Sakai K. Patel V. Fukumoto S. Yamada Y. (2014). Epiprofin orchestrates epidermal keratinocyte proliferation and differentiation. J. Cell Sci. 127, 5261–5272. 10.1242/jcs.156778 25344255 PMC4265740

[B50] Nieto M. A. Patel K. Wilkinson D. G. (1996). *In situ* hybridization analysis of chick embryos in whole mount and tissue sections. Methods Cell Biol. 51, 219–235. 10.1016/S0091-679X(08)60630-5 8722478

[B51] Phukan S. Babu V. S. Kannoji A. Hariharan R. Balaji V. N. (2010). GSK3beta: role in therapeutic landscape and development of modulators. Br. J. Pharmacol. 160, 1–19. 10.1111/j.1476-5381.2010.00661.x 20331603 PMC2860202

[B52] Ruch J. V. (1998). Odontoblast commitment and differentiation. Biochem. Cell Biol. 76, 923–938. 10.1139/o99-008 10392706

[B53] Sarkar L. Sharpe P. T. (2000). Inhibition of Wnt signaling by exogenous Mfrzb1 protein affects molar tooth size. J. Dent. Res. 79, 920–925. 10.1177/00220345000790040601 10831093

[B54] Sato N. Meijer L. Skaltsounis L. Greengard P. Brivanlou A. H. (2004). Maintenance of pluripotency in human and mouse embryonic stem cells through activation of Wnt signaling by a pharmacological GSK-3-specific inhibitor. Nature Med. 10, 55–63. 10.1038/nm979 14702635

[B55] Seidensticker M. J. Behrens J. (2000). Biochemical interactions in the Wnt pathway. Biochim. Biophys. Acta. 1495, 168–182. 10.1016/S0167-4889(99)00158-5 10656974

[B56] Shu W. Guttentag S. Wang Z. Andl T. Ballard P. Lu M. M.. (2005). Wnt/beta-catenin signaling acts upstream of N-myc, BMP4, and FGF signaling to regulate proximal-distal patterning in the lung. Dev. Biol. 283, 226–239. 10.1016/j.ydbio.2005.04.014 15907834

[B57] Siriwardena B. S. Kudo Y. Ogawa I. Tilakaratne W. M. Takata T. (2009). Aberrant beta-catenin expression and adenomatous polyposis coli gene mutation in ameloblastoma and odontogenic carcinoma. Oral Oncol. 45, 103–108 10.1016/j.oraloncology.2008.03.008 18486530

[B58] Thesleff I. Hurmerinta K. (1981). Tissue interactions in tooth development. Differentiation 18, 75–88. 10.1111/j.1432-0436.1981.tb01107.x 7011890

[B59] Thesleff I. Mikkola M. (2002). The role of growth factors in tooth development. Int. Rev. Cytol. 217, 93–135. 10.1016/S0074-7696(02)17013-6 12019566

[B60] Thesleff I. (2006). The genetic basis of tooth development and dental defects. Am J Med Genet. A. 140, 2530–2535. 10.1002/ajmg.a.31360 16838332

[B61] Unda F. J. Martín A. Hilario E. Bègue-Kirn C. Ruch J. V. Aréchaga J. (2000). Dissection of the odontoblast differentiation process in vitro by a combination of FGF1, FGF2, and TGFbeta1. Dev. Dyn. 218, 480–489. 10878613 10.1002/1097-0177(200007)218:3<480::AID-DVDY1011>3.0.CO;2-O

[B62] van Genderen C. Okamura R. M. Farinas I. Quo R. G. Parslow T. G. Bruhn L.. (1994). Development of several organs that require inductive epithelial-mesenchymal interactions is impaired in LEF-1-deficient mice. Genes Dev. 8, 2691–2703. 10.1101/gad.8.22.2691 7958926

[B63] Wang X. Suomalainen M. Jorgez C. Matzuk M. Werner S. Thesleff I. (2004). Follistatin regulates enamel patterning in mouse incisors by asymmetrically inhibiting BMP signaling and ameloblast differentiation. Dev. Cell. 7, 719–730. 10.1016/j.devcel.2004.09.012 15525533

[B64] Wang X. P. O'Connell D. J. Lund J. J. Saadi I. Kuraguchi M. Turbe-Doan A.. (2009). Apc inhibition of Wnt signaling regulates supernumerary tooth formation during embryogenesis and throughout adulthood. Development 136, 1939–1949. 10.1242/dev.033803 19429790 PMC2680115

[B65] Zmener O. (2009). Calcificación pulpar y endodoncia: estado actual, diagnóstico y posibilidades de tratamiento/Pulp calcification and endodontics: an approach to diagnosis and treatment possibilities. Rev. Asoc. Odontol. Argent. 97, 209–215.

