# Supplementary figures and images for: Wnt/β-Catenin Regulates the Activity of Epiprofin/Sp6, SHH, FGF, and BMP to Coordinate the Stages of Odontogenesis
Source: Front Cell Dev Biol. 2016 Mar 30;4:25. doi: 10.3389/fcell.2016.00025 (PMC4811915; doi:10.3389/fcell.2016.00025)

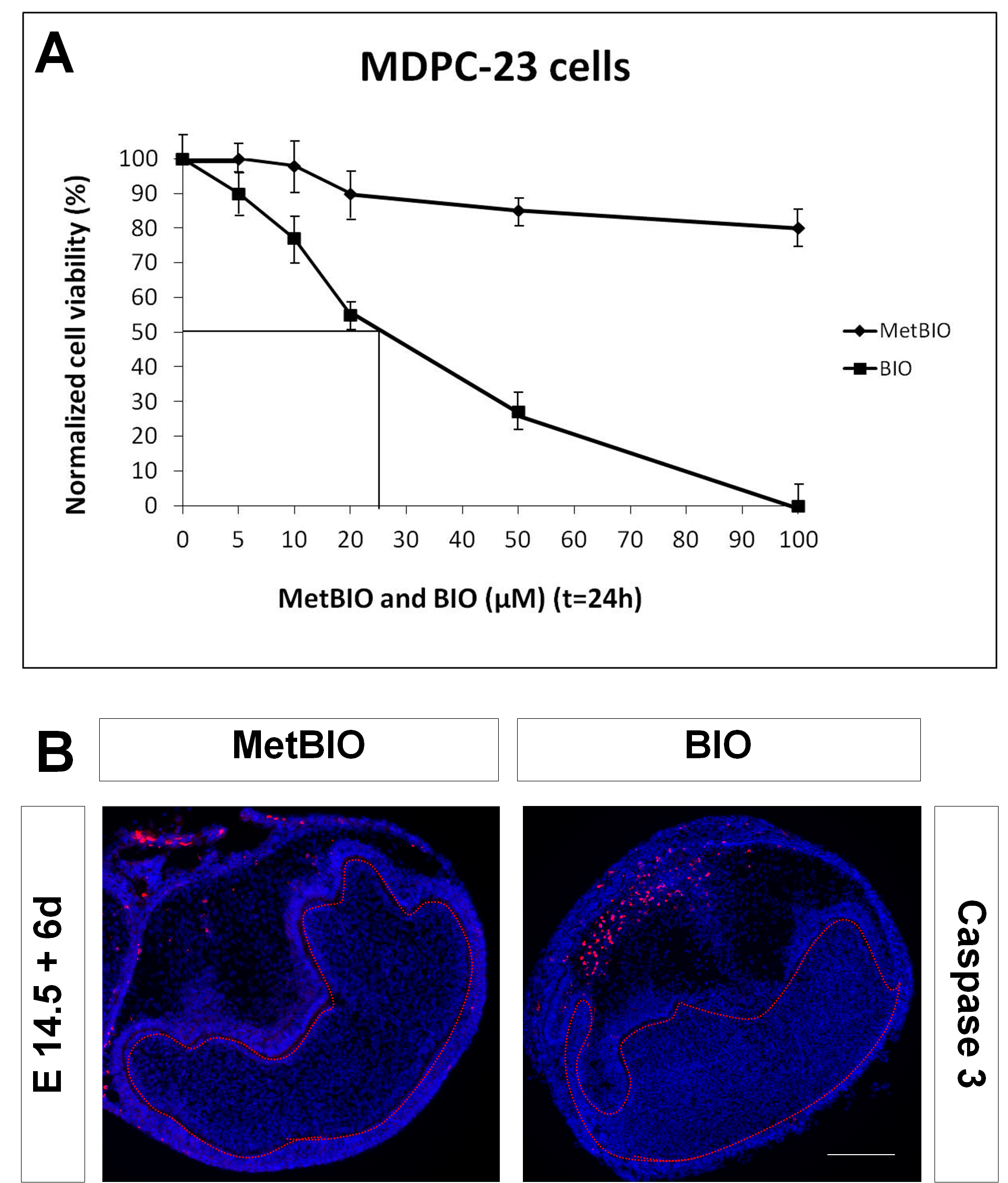

Supplement: Supplementary Figure 1 — Cell viabilty and apoptosis analysis. (A) XTT assay for cell viability was performed in MDPC-23 cells cultured with 0-100 μM MetBIO or BIO for 24 h. Effective concentration EC50 was determined at 25 μM BIO. (B) Immunofluorescence for Caspase3 in E14.5 molars cultured for 6 days in the presence of 20 μM MetBIO or BIO. Apoptotic cells were mainly detected in the oral epithelium. Only a few apoptosis appeared in the enamel organ of the tooth rudiments, both in control and treated samples. Red dots were drawn to show the frontier between dental epithelium and mesenchyme. Scale bars: 200 μm. [file Image1.TIF]
